# Charge Transfer and Charge Trapping Processes in Ca- or Al-Co-doped Lu_2_SiO_5_ and Lu_2_Si_2_O_7_ Scintillators Activated by Pr^3+^ or Ce^3+^ Ions

**DOI:** 10.3390/ma16124488

**Published:** 2023-06-20

**Authors:** Valentyn Laguta, Lubomir Havlak, Vladimir Babin, Jan Barta, Jan Pejchal, Martin Nikl

**Affiliations:** 1 Institute of Physics of the Czech Academy of Sciences, Cukrovarnicka 10/112, 16200 Prague, Czech Republic; 2 Faculty of Nuclear Sciences and Physical Engineering, Czech Technical University in Prague, Břehová 7, 11519 Prague, Czech Republic

**Keywords:** scintillation material, luminescence, EPR, lattice defect, radioluminescence

## Abstract

Lutetium oxyorthosilicate Lu_2_SiO_5_ (LSO) and pyrosilicate Lu_2_Si_2_O_7_ (LPS) activated by Ce^3+^ or Pr^3+^ are known to be effective and fast scintillation materials for the detection of X-rays and γ-rays. Their performances can be further improved by co-doping with aliovalent ions. Herein, we investigate the Ce^3+^(Pr^3+^) → Ce^4+^(Pr^4+^) conversion and the formation of lattice defects stimulated by co-doping with Ca^2+^ and Al^3+^ in LSO and LPS powders prepared by the solid-state reaction process. The materials were studied by electron paramagnetic resonance (EPR), radioluminescence spectroscopy, and thermally stimulated luminescence (TSL), and scintillation decays were measured. EPR measurements of both LSO:Ce and LPS:Ce showed effective Ce^3+^ → Ce^4+^ conversions stimulated by Ca^2+^ co-doping, while the effect of Al^3+^ co-doping was less effective. In Pr-doped LSO and LPS, a similar Pr^3+^ → Pr^4+^ conversion was not detected by EPR, suggesting that the charge compensation of Al^3+^ and Ca^2+^ ions is realized via other impurities and/or lattice defects. X-ray irradiation of LPS creates hole centers attributed to a hole trapped in an oxygen ion in the neighborhood of Al^3+^ and Ca^2+^. These hole centers contribute to an intense TSL glow peak at 450–470 K. In contrast to LPS, only weak TSL peaks are detected in LSO and no hole centers are visible via EPR. The scintillation decay curves of both LSO and LPS show a bi-exponential decay with fast and slow component decay times of 10–13 ns and 30–36 ns, respectively. The decay time of the fast component shows a small (6–8%) decrease due to co-doping.

## 1. Introduction

Scintillation materials are currently widely used for radiation detection in many fields, such as medical imaging, high energy physics calorimetry, bolometry for rare events searches, industrial control, safety and homeland security, and others [1]. Among them, wide bandgap oxide dielectrics with a high degree of structural perfection are the most suitable for such purposes [2]. In general, for scintillation applications, the material must accomplish fast and efficient transformations of incoming high energy photons/particles (or energy arising in a nuclear reaction with neutrons) into a number of electron–hole pairs collected in the conduction and valence bands, respectively, and their radiative recombination at suitable luminescence centers in the material. Therefore, most of the applications using scintillation materials are based on the density, scintillation, and time response performances. Based on these parameters, an impressive number of heavy cation-based hosts (particularly lutetium/yttrium/gadolinium) doped with Ce^3+^ or Pr^3+^ have been developed. Among them, a promising family of scintillation crystals is based on Lu_2_SiO_5_ (LSO), (Lu,Y)_2_SiO_5_ (LYSO) oxyorthosilicates, and Lu_2_Si_2_O_7_ (LPS) and recently (Gd,La)_2_Si_2_O_7_ pyrosilicates doped by Ce^3+^ or Pr^3+^ ions (see, e.g., review paper [2] and refs. [3,4,5,6]). All the abovementioned materials are excellent candidates for the detection of gamma rays in both positron emission tomography (PET), a very powerful medical imaging method to monitor metabolism, blood flow, or neurotransmission [7], and high energy calorimetry [2,8]. LYSO:Ce crystals are currently used in scintillation detectors in PET scanners, and various co-doping schemes have been reported in the last decade to further improve their performance [2,9,10]. In particular, co-doping LSO:Ce with divalent Ca^2+^ or Mg^2+^ ions has been shown to eliminate shallow electron traps and decrease the scintillation decay time from ~43 ns to ~30 ns while maintaining a high light output [8,11,12]. This improvement is at least partially related to the effective Ce^3+^ → Ce^4+^ conversion, where the stable Ce^4+^ ion creates an additional fast radiative recombination pathway, which efficiently competes in electron trapping from the conduction band with any other electron traps [11]. Alternatively, co-doping with a trivalent metal ion (Al, Ga, or In) that substitutes a tetravalent Si ion has been proposed [13,14]. It was assumed that such co-doping creates a positive charge deficit that limits the trapping of electrons responsible for afterglow.

The same positive effect of Ca^2+^(Mg^2+^) or Al^3+^ co-doping on decay time improvements is expected in Ce- or Pr-doped Lu_2_Si_2_O_7_ pyrosilicates [15]. They show even better scintillation characteristics than oxyorthosilicates. In particular, with nearly the same light yield, energy resolution, and scintillation decay time as reported for LSO:Ce, Ce-doped pyrosilicates are free from the intense afterglow as reported in [16,17]. However, in general, the effect of Ca^2+^, Mg^2+^, or Al^3+^ co-doping on charge trapping processes has not been investigated practically, especially in Pr-doped oxyorthosilicates and pyrosilicates.

In oxyorthosilicates as well as in scintillating garnets, in addition to the Ce^3+^ → Ce^4+^ recharge that improves the timing characteristics, the creation of additional charge trapping sites due to co-doping with aliovalent ions is important as well. Such traps will decrease the light yield and can also contribute to delayed luminescence or afterglow. This phenomenon can seriously limit the time response of a scintillator, which is crucial for PET scanners and other time-of-flight applications where a sub-100 ps time resolution is desirable. Although charge traps have been extensively studied in both LSO and LPS activated by Ce^3+^ and Pr^3+^ ions using the thermally stimulated luminescence (TSL) and the local probe electron paramagnetic resonance (EPR) methods [18,19,20,21], not much is known about charge traps in Ca^2+^(Mg^2+^) or Al^3+^ co-doped materials.

In this paper, we present the results of a detailed EPR study of polycrystalline Ce- and Pr-doped Lu_2_SiO_5_ and Lu_2_Si_2_O_7_ co-doped with Ca^2+^ and Al^3+^ with the aim of clarifying the Ce^3+^(Pr^3+^) → Ce^4+^(Pr^4+^) conversion and to study the formation of lattice defects stimulated by co-doping with aliovalent ions. Our EPR study is also accompanied by the TSL and scintillation decay measurements of the synthesized materials.

## 2. Materials and Methods

The powder samples of LSO and LPS doped with 2000 ppm Ce (or Pr)/Lu and co-doped with 5000 ppm Al/Si or 5000 ppm Ca/Lu (see Table 1) were prepared by a conventional solid-state reaction process [22] consisting of several periods of annealing in air up to 1500–1600 °C/72 h and remixing in an agate mortar. The starting materials were 5N Lu_2_O_3_, 4N8 SiO_2_, 5N Al_2_O_3_, CaO grade I, 4N CeO_2_, or 5N Pr_6_O_11_. The weights of the starting oxides were corrected for the moisture content in the base materials determined after annealing at 1200 °C/12 h. Annealing was carried out in corundum boats with lids, which were washed and then annealed up to 1600 °C/12 h before their first use. It should be noted that due to segregation during the growth of crystallites, the final contents of the dopants in the lattice could be smaller by up to a factor of 0.5 [8] than those indicated in Table 1.

The phase purity of all synthesized LSO and LPS powders was evaluated by X-ray powder diffraction (XRPD) using a Rigaku MiniFlex 600 diffractometer equipped with a Cu X-ray tube and a NaI:Tl scintillation detector. The diffraction patterns exactly matched the following LSO and LPS database records in the ICDD PDF-2 database: 01-070-9485 and 01-071-3309, respectively (see Supplementary Materials). This proved that all samples after the final annealing step were phase-pure materials with a phase composition corresponding to the desired one; LSO samples contained just Lu_2_SiO_5_ (space group *C*2/*c* [23]) and LPS samples contained just Lu_2_Si_2_O_7_ (space group *C*2/*m* [24]).

EPR spectra were measured using a commercial Bruker EMX plus spectrometer at X-band (microwave frequency 9.25–9.5 GHz) within the temperature range of 10–290 K. An X-ray tube operating at a voltage and current of 55 kV and 30 mA, respectively, with a Co anode (ISO-DEBYEFLEX 3003 Seifert Gmbh., Ahrensburg, Germany) was used as the source of X-ray irradiation for LSO and LPS powders.

The radioluminescence (RL) and thermally stimulated luminescence measurements were performed in the spectral range 200–800 nm using the Horiba Jobin-Yvon 5000M spectrometer with an Oxford liquid nitrogen cryostat and a TBX-04 (IBH) photomultiplier (Glasgow, Scotland). The spectral bandwidth of the monochromator was 8 nm. The RL spectra were recorded at 295 K. The spectrally unresolved TSL glow curves were recorded in the temperature range of 77–500 K with a heating rate of 0.1 K/s. Irradiation of the samples was performed at 77 K via a Seifert X-ray tube operating at 40 kV with a tungsten target; the dose was estimated to be about 450 Gy. All the spectra were corrected for spectral distortions caused by the experimental setup.

Scintillation decays with ultra-high time resolution under the pulsed X-ray excitation were measured using a picosecond (ps) X-ray tube N5084 (Hamamatsu, 40 kV, Shizuoka, Japan). The X-ray tube was driven by a ps pulsed laser at a repetition rate of up to 1 MHz. The signal was detected by a hybrid picosecond photon detector and a Fluorohub unit (Horiba Scientific). The instrumental response function FWHM of the setup is about 75 ps. Spectrally unresolved luminescence decay curves were detected from the surface excited by X-rays. The convolution procedure of the instrumental response and fit function was applied to fit the decay curves and determine the true decay times (SpectraSolve^TM^ software package for Windows, Ames Photonics, Hurst, TX, USA).

## 3. Results

### 3.1. EPR Spectra in Ca and Al Co-doped LSO:Ce and LSO:Pr

**Figure 1:**
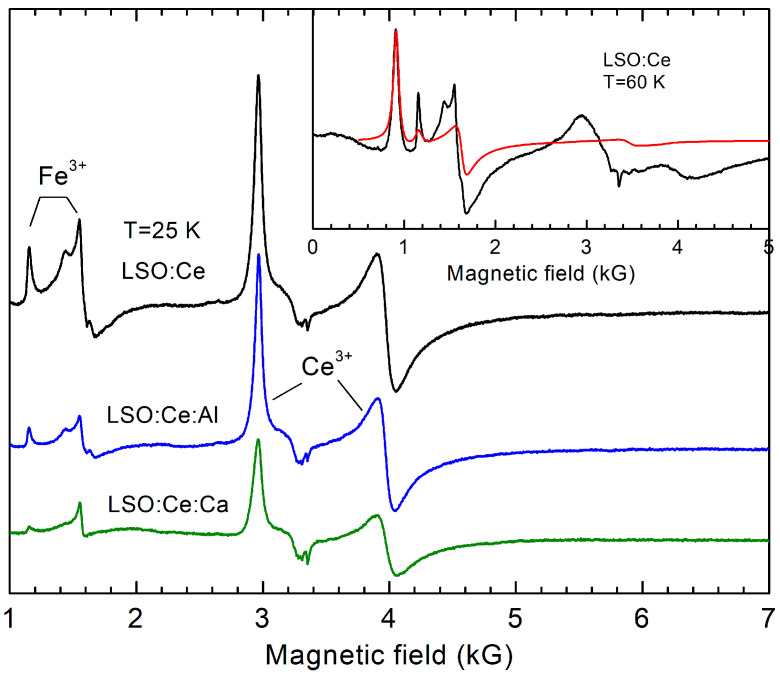
EPR spectra measured in LSO:Ce showing the change in the Ce^3+^ and Fe^3+^ concentration (EPR intensity is directly proportional to concentration) under co-doping with Ca^2+^ and Al^3+^ ions. The signal at low magnetic fields is assigned to Fe^3+^ ions. The inset shows a comparison of the Fe^3+^ simulated spectrum (red line) with the measured spectrum (black line). All spectra are normalized to the same sample volume.

A detailed investigation of the Ce^3+^ → Ce^4+^ charge conversion that plays an important role in the acceleration of the Ce^3+^ scintillation decay was performed on LSO:Ce, LSO:Ce,Al, and LSO:Ce,Ca samples. The corresponding EPR spectra are presented in Figure 1. The spectra contain two spectral lines from Ce^3+^ ions (*S* = 1/2, 4*f*^1^) corresponding to two principal *g* factors: *g*_1_ = 2.262 and *g*_2_ = 1.686. The third Ce^3+^ spectral line at *g*_3_ = 0.563 (*B*_r_ = 16.676 kG) is outside of the magnetic field range. These *g* factors coincide with those measured previously in crystals [25]. Note that the second Ce^3+^ center detected in crystals was not resolved in the powder spectrum due to the low intensity of its spectral lines (the population of the second Ce^3+^ center is only about 5% [25]).

There are other spectral lines at low magnetic fields, which we assign to Fe^3+^ accidental impurities. The spectrum is typical for this ion (*S* = 5/2, 3*d*^5^) in a low-symmetry crystal field with large zero-field splitting of energy levels [26]. The corresponding simulated spectrum is shown in the inset of Figure 1 (red line), and the spin Hamiltonian parameters are listed in Table 2. For the fit, the conventional spin Hamiltonian was used:

$$H=\beta S\cdot g\cdot B+\frac{1}{3}(b_{2}^{0}O_{2}^{0}+b_{2}^{2}O_{2}^{2}),$$

where *β* is the Bohr magneton, **g** is the *g*-tensor, and $b_{i}^{m}$ and $O_{l}^{m}$ are crystal field parameters and Stevens spin operators [27], respectively. The fourth-rank crystal field terms were neglected as they are much smaller than the other terms and could not be determined from powder spectra. The roughly estimated concentration of Fe^3+^ ions from the EPR intensity is only 5–10 ppm. These ions, most probably, come from the raw materials.

A substantial (three-fold) decrease in Ce^3+^ concentration in Ca-co-doped samples was observed, confirming the effective Ce^3+^ → Ce^4+^ conversion, while the effect of Al co-doping (when Al^3+^ is substituted for Si^4+^) was much smaller, at only about 15–20%. The Fe^3+^ EPR intensity also decreases under Ca and Al co-doping, indicating the recharge of the Fe^3+^ ions to a valence state invisible in EPR.

**Figure 2:**
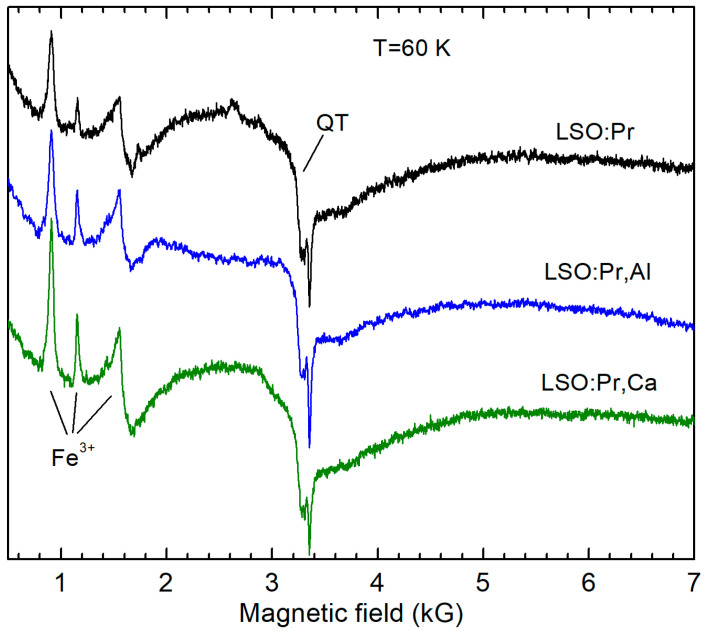
EPR spectra measured in LSO:Pr, LSO:Pr,Al, and LSO:Pr,Ca powders showing the change in the Fe^3+^ concentration under Al and Ca co-doping. The broad signal denoted as QT belongs to quartz tubes of LHe cryostat. All spectra are normalized to the same sample volume.

We also measured the Pr^3+^ → Pr^4+^ conversion in LSO:Pr samples stimulated by Al and Ca co-doping. The EPR spectrum of Pr^3+^ (*S* = 1, 4*f*^2^) is not visible at 9–35 GHz which is available in our spectrometer due to a large splitting of the 4*f*^2^ energy levels already in zero field (non-paramagnetic ground state is not excluded as well). Moreover, even Pr^4+^, which has the same electron shell as Ce^3+^ and should be easily detectable in EPR, was not detected in the EPR spectra even at measurement temperatures down to 3.5 K. This suggests a much lower concentration of Pr^4+^ ions compared to Pr^3+^ ions. Therefore, the Pr^3+^ → Pr^4+^ conversion stimulated by Al and Ca co-doping of LSO is much less effective than the Ce^3+^ → Ce^4+^ conversion. Instead, as in the case of LSO:Ce, we observed a pronounced change in the Fe^3+^ concentration (Figure 2) induced by Al and Ca co-doping of LSO:Pr. However, here, the Fe^3+^ concentration increases, suggesting that the Fe^2+^ → Fe^3+^ conversion takes place to compensate, at least partly, the excess negative charge caused by replacement of Lu^3+^ by Ca^2+^.

### 3.2. EPR Spectra in Ca- and Al-Co-doped LPS:Ce and LPS:Pr

**Figure 3:**
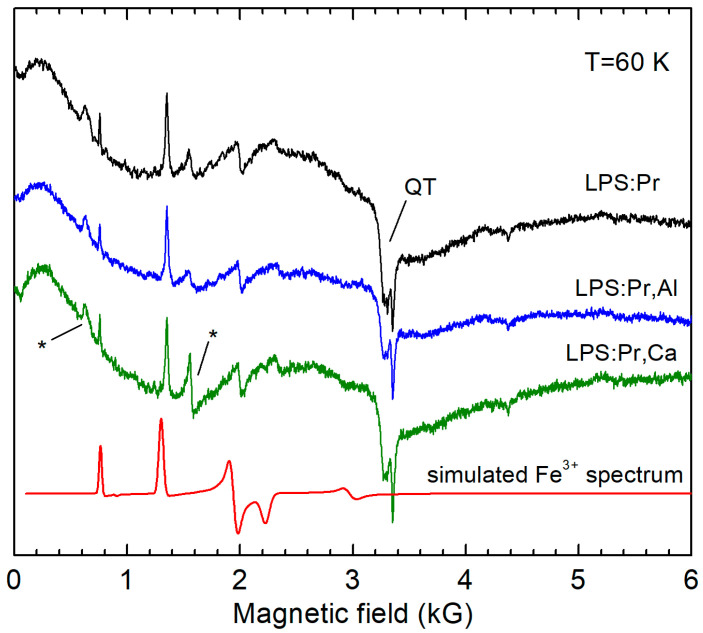
EPR spectra measured in LPS:Pr, LPS:Pr,Al, and LPS:Pr,Ca powders. The broad signal denoted as QT belongs to quartz tubes of the LHe cryostat and asterisks denote unidentified spectral lines. The simulated Fe^3+^ spectrum (red line) is shown as well. All spectra are normalized to the same sample volume.

**Figure 4:**
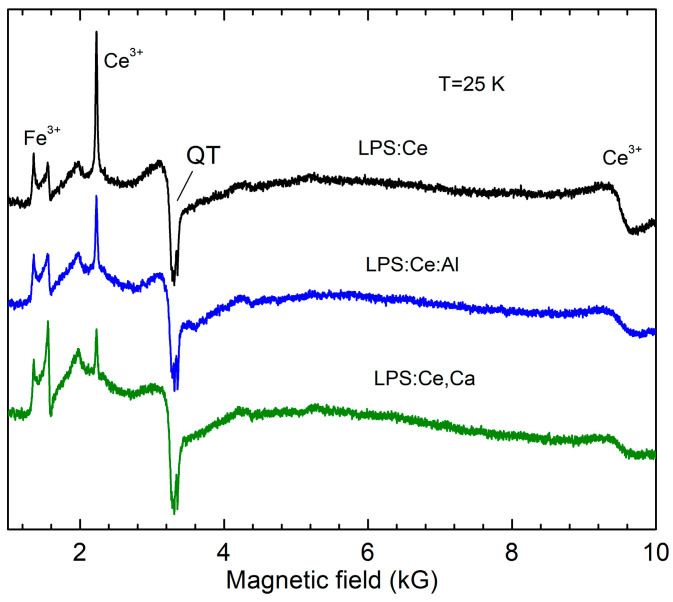
EPR spectra measured in LPS:Ce showing the change in the Ce^3+^ concentration under co-doping with Ca^2+^ and Al^3+^ ions. All spectra are normalized to the same sample volume.

The EPR spectra measured in LPS:Pr, LPS:Pr,Al, and LPS:Pr,Ca powders are presented in Figure 3. As in the case of Pr-doped LSO samples, only Fe^3+^ ions were detected in EPR spectra. The spectral parameters of these ions in the LPS lattice (obtained from the simulation of the Fe^3+^ powder spectrum) are listed in Table 2. In contrast to the Pr-doped LSO, no change in the Fe^3+^ concentration was observed in LPS. However, in Ce-doped LPS (Figure 4), the Fe^3+^ concentration markedly increased with Ca co-doping. The Ce^3+^ spectral lines were identified according to the data published in [25,28]. The Ce^3+^ concentration markedly decreases with Al and Ca co-doping. The effect of the co-doping is especially large for the Ca co-dopant, similar LSO:Ce, indicating an effective Ce^3+^ → Ce^4+^ conversion.

### 3.3. EPR Spectra Created by X-ray Irradiation in LPS:Ce, LPS:Pr, LSO:Ce, and LSO:Pr Co-doped with Ca^2+^ or Al^3+^ and Comparison with TSL

**Figure 5:**
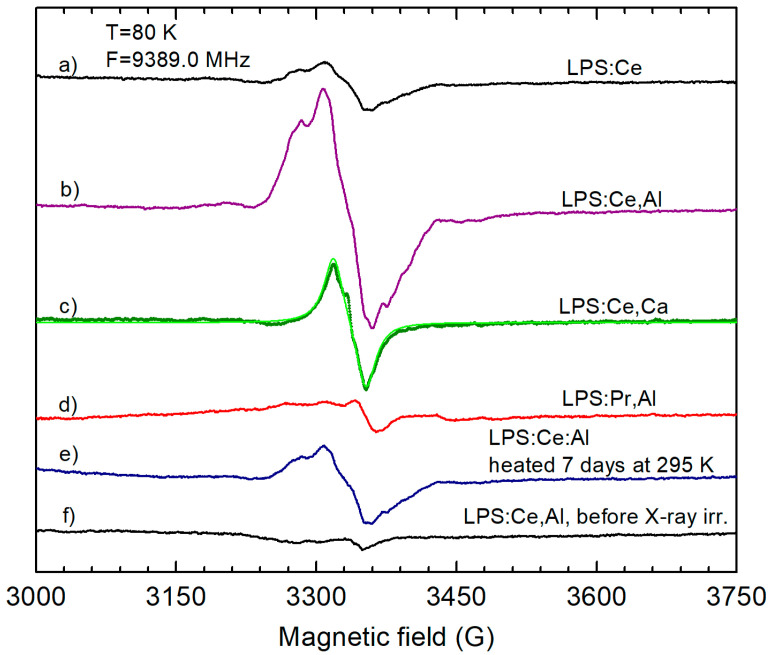
O^−^ EPR spectra created by X-ray irradiation at room temperature in (**a**) LPS:Ce; (**b**) LPS:Ce,Al; (**c**) LPS:Ce,Ca; and (**d**) LPS:Pr,Al. (**e**) EPR spectrum after heating of the X-ray irradiated LPS:Ce,Al powder at 295 K for 7 days. (**f**) EPR spectrum measured in LPS:Ce,Al before X-ray irradiation. Simulated O^−^ EPR spectrum is shown by the green solid line for LPS:Ce,Ca in spectrum (**c**). The simulated spectrum coincides with the measured one (black solid line).

The effect of Al and Ca co-doping on the hole and electron trapping processes was investigated in X-ray-irradiated LPS:Ce(Pr) and LSO:Ce(Pr) samples via EPR measurements of X-ray-irradiation-induced paramagnetic centers and with TSL measurements. These measurements showed formation of O^−^ hole centers (a hole trapped at an oxygen ion) stabilized by Al^3+^ or Ca^2+^ ions at Si^4+^ or Lu^3+^ sites in LPS samples, respectively. Figure 5 illustrates the O^−^ EPR spectra created by X-ray irradiation at 295 K for selected samples.

The interpretation of these O^−^ spectra is based on our detailed study of the O^−^ centers in LPS:Ce and LPS:Pr crystals [21]. In particular, the O^−^ EPR spectra are mainly constructed from unresolved hyperfine components from ^175^Lu isotopes (nuclear spin *I* = 7/2, 97.4% natural abundance). In single crystal samples, the hyperfine structure of ^175^Lu isotopes is clearly observed [21]. As can be seen from Figure 5a,b, X-ray irradiation creates the same spectrum in both LPS:Ce and LPS:Ce,Al samples. However, the spectral intensity is much higher in the LPS:Ce,Al sample, suggesting that O^−^ centers are created near Al^3+^ impurities (Al^3+^ at Si^4+^ sites stabilizes the trapped hole in the neighboring oxygen lattice ion). Such hole centers are usually called bound small polarons [29]. The nominally Al undoped LPS:Ce probably also contains small amounts of the same O^−^–Al centers, as powders were synthesized in an Al_2_O_3_ boat at 1500–1600 °C. It is assumed that the trapped hole interacts with the nuclear spins of the two nearest Lu ions and the nuclear spin of Al impurities. Together with the *g* factor anisotropy, this creates quite a complex spectral pattern which cannot realistically be simulated due to many unknown parameters. Therefore, this center was characterized in this work only by its average *g* factor value measured at the center of gravity of the spectral line (Table 2).

In the case of Ca co-doping, the Ca^2+^ ion at the Lu^3+^ site also serves as a stabilizing defect for the hole trapped at an oxygen ion. In the O^−^–Ca centers, trapped holes interact only with the nuclear spin of one Lu ion (Ca has no isotopes with non-zero nuclear spins and the ^29^Si isotope only has a small natural abundance of 4.67%) and the O^−^ spectral line in the LPS:Ce,Ca sample is narrow (Figure 5c). Its shape can be easily simulated including the ^175^Lu hyperfine interaction (green solid line in Figure 5c and parameters in Table 2).

**Figure 6:**
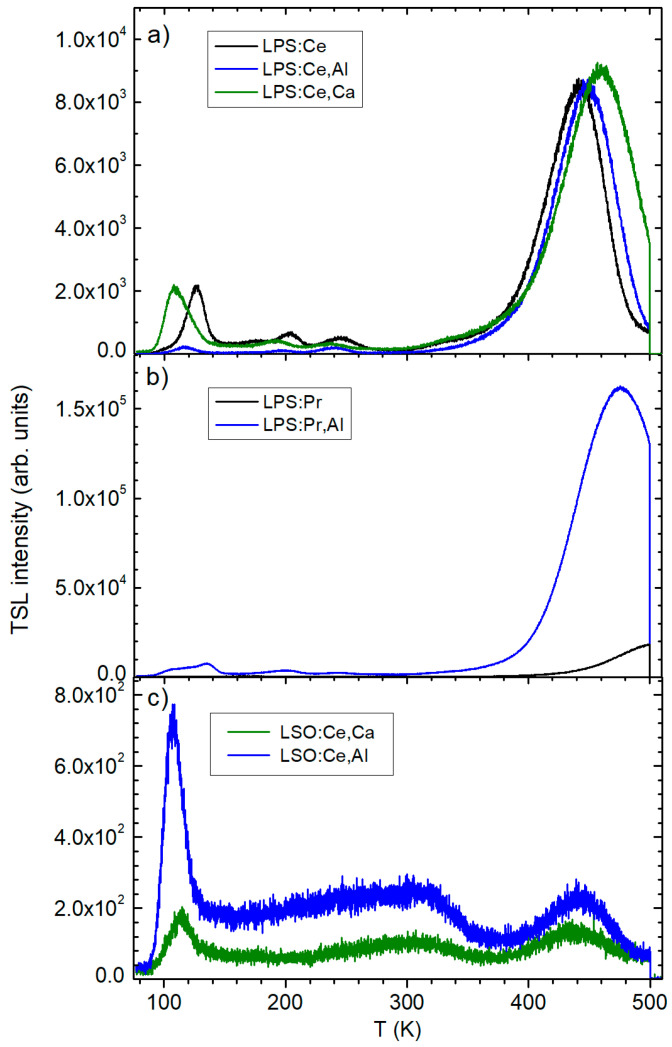
TSL glow curves of: (**a**) LPS:Ce, LPS:Ce,Al, and LPS:Ce,Ca; (**b**) LPS:Pr and LPS:Pr,Al; and (**c**) LSO:Ce,Al and LSO:Ce,Ca. TSL is created by X-ray irradiation at 77 K.

X-ray irradiation of Pr-doped LPS also created O^−^ hole centers. However, their EPR spectrum is broad (Figure 5d) and could not be qualitatively analyzed. O^−^ centers in LPS created by X-ray irradiation at room temperature are thermally stable to about 350 K, but their concentration slowly decreases even at room temperature (Figure 5e). They completely disappear at annealing temperatures of ~450–500 K (see also ref. [21]). This correlates well with the main TSL peak at 440–480 K created by X-ray irradiation measured in the same samples (Figure 6a,b). This TSL peak is broad and contains contributions from several traps [19,30], not all of them being paramagnetic. A complete fitting of the TSL peaks is presented in refs. [19,20,30]. Therefore, in general, the intensity of TSL does not correlate exactly with the intensity of the EPR spectra. On the other hand, one can notice that the TSL intensity is much higher in the Al-co-doped LPS:Pr sample as compared to the Al-free sample (Figure 6b).

Surprisingly, no X-ray-created paramagnetic active centers were detected in LSO samples even after irradiation at 77 K. TSL measurements revealed a weak glow peak at ≈110 K (Figure 6c), indicating an effective (practically full) recombination of electron–hole pairs at activator ions. This contradicts the data obtained in measurements of LSO:Ce single crystals, where a sequence of intense glow peaks was observed at 350–600 K [11,20]. To explain the nature of the corresponding charge traps, the authors of ref. [20] proposed a model where Ce^3+^ serves as the charge donor, the recombination center, and also the trap creating center. The traps are related to specific configurations of oxygen ions around the central Ce^3+^ ion. Each configuration is able to trap the 5d electron in a metastable electronic state, with mixed Ce^3+^ 5d and O^2−^ orbitals at the same time creating a hole state near or at the Ce ion. According to our EPR data, the hole state is the O^−^ ion rather than the Ce^4+^ ion, but both may exist depending on the presence of other defects in the surrounding lattice.

### 3.4. Scintillation Decay Time Measurements

**Figure 7:**
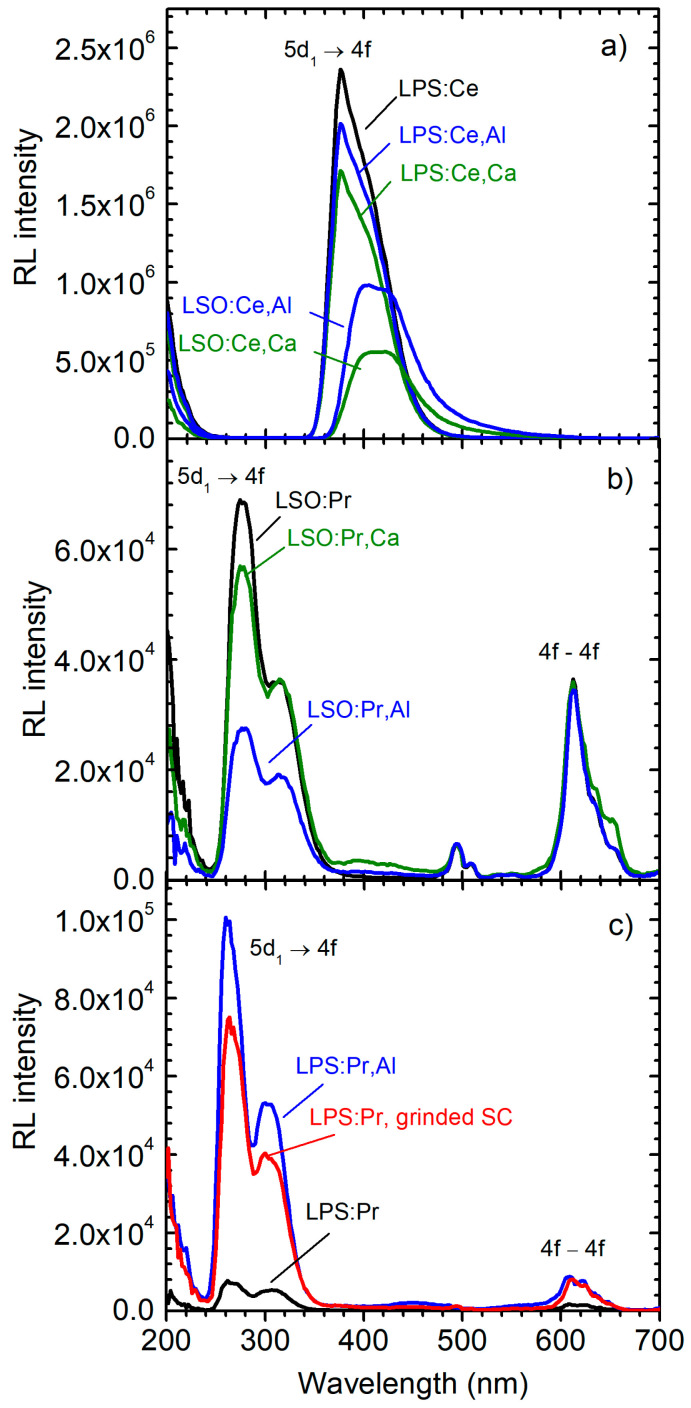
X-ray excited luminescence spectra measured at 296 K in (**a**) LPS:Ce, LPS:Ce,Al, LPS:Ce,Ca, LSO:Ce,Al, and LSO:Ce:Ca powders; (**b**) LSO:Pr, LSO:Pr,Ca, and LSO:Pr,Al powders; and (**c**) LPS:Pr and LPS:Pr,Al powders and LPS:Pr grinded SC.

For selected samples, radioluminescence and scintillation decay curves within two time windows of 50 ns and 2 μs were measured at 296 K. The emission spectra of LPS:Ce samples (Figure 7a) are characterized by intense Ce-related bands with maxima located at about 380 nm in all samples. The emission intensity is largest in the non-co-doped sample and decreases by about 1.2-fold in the Al-co-doped sample and by 1.4-fold in the Ca-co-doped sample. In LSO:Ce samples, the Ce-related emission bands have maxima at about 405 nm; the intensity is the highest in the Al-co-doped sample while that of the Ca-co-doped it is about 2 times lower.

The spectra of LSO:Pr (Figure 7b) are dominated by the 5d–4f doublet emission peak with maxima at about 280 nm. Weaker 4f–4f transitions can be observed at longer wavelengths at around 610 nm. The emission spectra of LPS:Pr samples (Figure 7c) are similar to those of LSO:Pr, but the main 5d–4f emission peak is shifted to 260 nm and the 4f–4f transitions are much weaker. Although co-doping does not influence the spectral position of the emission bands, it markedly changes the RL emission intensity.

**Figure 8:**
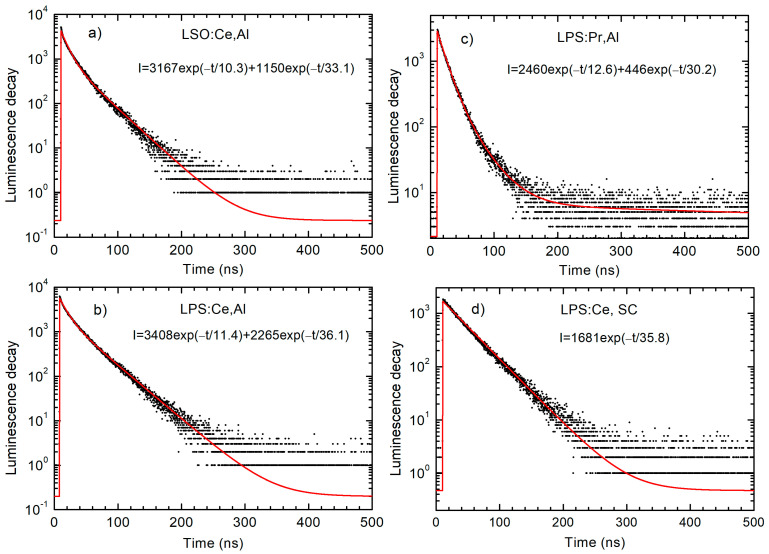
Luminescence decay of Ce^3+^ and Pr^3+^ emissions excited by X-ray pulses at 296 K in (**a**) LSO:Ce,Al, (**b**) LPS:Ce,Al, and (**c**) LPS:Pr,Al powder samples. For comparison, the luminescence decay in the LPS:Ce single crystal grinded in powder is presented in graph (**d**). The fit of the experimental data (black dots) is shown by the red solid lines.

In order to study the influence of co-doping on scintillation kinetics characteristics, the ps-pulsed X-ray excited decay curves were measured with an ultra-high time resolution, and the results are shown in Figure 8.

All experimental decay curves were fitted using a bi-exponential function and the calculated parameters are presented in Table 3. The measurements showed, along with the conventional 33–38 ns decay component in the Ce-doped samples, a second fast component with the decay time of only about 11 ns. On the contrary, in the Pr-doped samples, along with the conventional fast decay component 13–14 ns, a second slower component (30–31 ns) is presented. The origin of the second components is unclear. To the best of our knowledge, the fast 11 ns scintillation decay component in Ce-doped LSO and LPS has never been observed before and this result needs further detailed investigation. For comparison, the decay curve of the LPS:Ce single crystal grinded into a powder can be fitted using a single exponential function (Figure 8d).

The changes in the decay time values due to co-doping are almost negligible. However, some common tendencies could be observed. The values of both fast and slow decay components demonstrate a mild decrease with the following sequence: non-co-doped → Al-co-doped → Ca-co-doped samples (e.g., compare τ_1_ component in LPS:Ce: 11.5 → 11.4 → 10.8 ns).

## 4. Conclusions

Single-phase powder samples of Lu_2_SiO_5_ (space group *C*2/*c*) and Lu_2_Si_2_O_7_ (space group *C*2/*m*) doped with 2000 ppm Ce (or Pr) and co-doped with 5000 ppm Al/Si or 5000 ppm Ca/Lu were prepared by the conventional solid-state reaction process. The phase purity was confirmed by X-ray diffraction measurements.

Detailed EPR measurements of LSO:Ce revealed a substantial (three-fold) decrease in the Ce^3+^ concentration in Ca-co-doped samples, confirming the effective Ce^3+^ → Ce^4+^ conversion, while the effect of Al co-doping (Al^3+^ substituted for Si^4+^) was much smaller, at only a 15–20% decrease.

On the contrary, EPR measurements in LPS:Ce, LPS:Ce,Al, and LPS:Ce,Ca samples revealed approximately the same decrease in Ce^3+^ concentration (2–3 times) in both the Al- and Ca-co-doped samples (again with a stronger effect for Ca-co-doped sample), suggesting that the crystal structure and different number of chemical bonds with oxygen ions in LSO and LPS influences the charge balance and charge transfer between doping ions.

In both LSO and LPS samples, accidental Fe^3+^ impurities were detected by EPR. The Fe^3+^ EPR intensity changed under Ca and Al co-doping, indicating participation of the Fe^3+^ ions in the charge transfer processes induced by Ca and Al co-doping.

EPR measurements in the Pr-doped LSO and LPS did not reveal possible Pr^3+^ → Pr^4+^ conversions stimulated by Al and Ca co-doping, despite the fact that the Pr^4+^ ion has the same electron shell as Ce^3+^ and, consequently, it should be clearly observed by EPR. This suggests that the charge compensation of Al^3+^ and Ca^2+^ ions is mainly realized via participation of other impurities and/or lattice defects.

X-ray irradiation of Ce- and Pr-doped LPS creates O^−^ centers attributed to a hole trapped at an oxygen ion in the neighborhood of Al^3+^ and Ca^2+^ ions. These hole centers are reasonably thermally stable up to about 400 K. They contribute to intense TSL glow peaks at 450–470 K. On the other hand, only a weak TSL peak was visible in all LSO samples at T ≈ 110 K and no paramagnetic active centers were detected via EPR after X-ray irradiation despite the intense radioluminescence. This suggests effective (practically full) recombination of electron–hole pairs at activator ions.

The X-ray-excited scintillation decay curves were measured for both LSO and LPS samples and could be approximated by a bi-exponential function. The measurements showed, along with the conventional dominating 33–38 ns decay component in Ce-doped LSO and LPS, the presence of a second fast component with a decay time of only about 11 ns. The origin of the latter component is unclear and needs further detailed investigations. On the contrary, in the Pr-doped samples, along with the usual fast decay component of 13–15 ns, a second slower component of 30–31 ns is present. Only a weak acceleration of the scintillation decay (6–8%) is found for the fast component in the Ca- and Al-co-doped samples. For the slower component, the acceleration is negligibly small.

## Figures and Tables

**Table 1:** Nominal composition of samples.

Theoretical Sample Formula	Designation in the Text
Lu_1_._996_Ce_0_._004_SiO_5_	LSO:Ce
Lu_1_._986_Ce_0_._004_Ca_0_._01_SiO_5_	LSO:Ce,Ca
Lu_1_._996_Ce_0_._004_Si_0_._995_Al_0_._005_O_5_	LSO:Ce,Al
Lu_1_._996_Ce_0_._004_Si_2_O_7_	LPS:Ce
Lu_1_._986_Ce_0_._004_Ca_0_._01_Si_2_O_7_	LPS:Ce,Ca
Lu_1_._996_Ce_0_._004_Si_1_._99_Al_0_._01_O_7_	LPS:Ce,Al
Lu_1_._996_Pr_0_._004_SiO_5_	LSO:Pr
Lu_1_._986_Pr_0_._004_Ca_0_._01_SiO_5_	LSO:Pr,Ca
Lu_1_._996_Pr_0_._004_Si_0_._995_Al_0_._005_O_5_	LSO:Pr,Al
Lu_1_._996_Pr_0_._004_Si_2_O_7_	LPS:Pr
Lu_1_._986_Pr_0_._004_Ca_0_._01_Si_2_O_7_	LPS:Pr,Ca
Lu_1_._996_Pr_0_._004_Si_1_._99_Al_0_._01_O_7_	LPS:Pr,Al

**Table 2:** Spectral parameters of Ce^3+^, Fe^3+^, and O^−^ centers in LSO and LPS powders.

Material	Spin Hamiltonian Parameters	HF Constant	Reference
LSO:Ce LSO:Pr	Ce^3+^: *g*_1_ = 2.262 *g*_2_ = 1.686 *g*_3_ = 0.563 Fe^3+^: *g* = 1.99 $b_{2}^{0}$ = 0.165 cm^−1^ $b_{2}^{2}$ = 0.165 cm^−1^		This paper, [25]
			This paper

LPS:Ce LPS:Pr	Ce^3+^: *g*_1_ = 3.000 *g*_2_ = 0.705 *g*_3_ ≈ 0.10 Fe^3+^: *g* = 2.00 $b_{2}^{0}$ = 0.700 cm^−1^ $b_{2}^{2}$ = 0.380 cm^−1^		This paper, [25,28]
			This paper

LPS:Ce,Al	O^−^: *g* = 2.012	Not determined	This paper
LPS:Ce,Ca	O^−^: *g* = 2.011	^175^Lu: A_00_ = 4.0 × 10^−4^ cm^−1^	This paper

**Table 3:** Luminescence decay times and relative intensities of components in LPS and LSO powders doped with Ce or Pr and co-doped with Ca or Al, approximated by the bi-exponential function $I(t)=\sum A_{i}\exp(-t/\tau_{i})+b,$ where *i* = 1, 2, to fit the scintillation decay curves. The relative intensity of each component is calculated as $I_{i}=(A_{i}\tau_{i}/\sum A_{i}\tau_{i})\times 100\%.$ For comparison, the luminescence decay time of the LPS:Ce single crystal and published data for decay times in LSO and LPS under γ-ray excitation are listed as well.

Composition	τ_1_ (ns)	*I*_1_ (%)	τ_2_ (ns)	*I*_2_ (%)
LPS:Ce	11.5	31.3	36.3	68.7
LPS:Ce,Al	11.4	32.2	36.1	67.8
LPS:Ce,Ca	10.8	32.5	35.5	67.5
LPS:Ce, SC			35.8, 38 *	100
LSO:Ce,Al	10.3	46.1	33.1	53.9
LSO:Ce,Ca	9.8	50.8	32.9	49.2
LSO:Ce, SC			40 *	100
LPS:Pr	13.6	69.0	30.8	31.0
LPS:Pr,Al	12.6	69.7	30.2	30.3
LPS:Pr, SC	20 **, 15 ***	100		

* Decay time in SC under γ-ray excitation [16]; ** photoluminescence decay time in SC under excitation at 256 nm [28]; *** decay time in SC under γ-ray excitation [31].

## Data Availability

Data are contained within the article.

## Supplementary Materials

The following supporting information can be downloaded at: https://www.mdpi.com/article/10.3390/ma16124488/s1.
