# Supplementary material for: Charge Transfer and Charge Trapping Processes in Ca- or Al-Co-doped Lu2SiO5 and Lu2Si2O7 Scintillators Activated by Pr3+ or Ce3+ Ions
Source: Materials (Basel). 2023 Jun 20;16(12):4488. doi: 10.3390/ma16124488 (PMC10302549; doi:10.3390/ma16124488)
Supplement: Supplementary file 1 [file materials-16-04488-s001.zip › materials-2424146-supplementary.pdf]

Charge Transfer and Charge Trapping Processes in Ca- or Al-Co-doped Lu_2SiO_5 and $Lu_2Si_2O_7$ Scintillators Activated by Pr^{3+} or Ce^{3+} Ions.

Valentyn Laguta, Lubomir Havlak, Vladimir Babin, Jan Barta, Jan Pejchal and Martin Nikl

Supplementary information

<u>Measured X-ray diffraction patterns compared to ICDD PDF-2 database records (ver. 2013)</u>

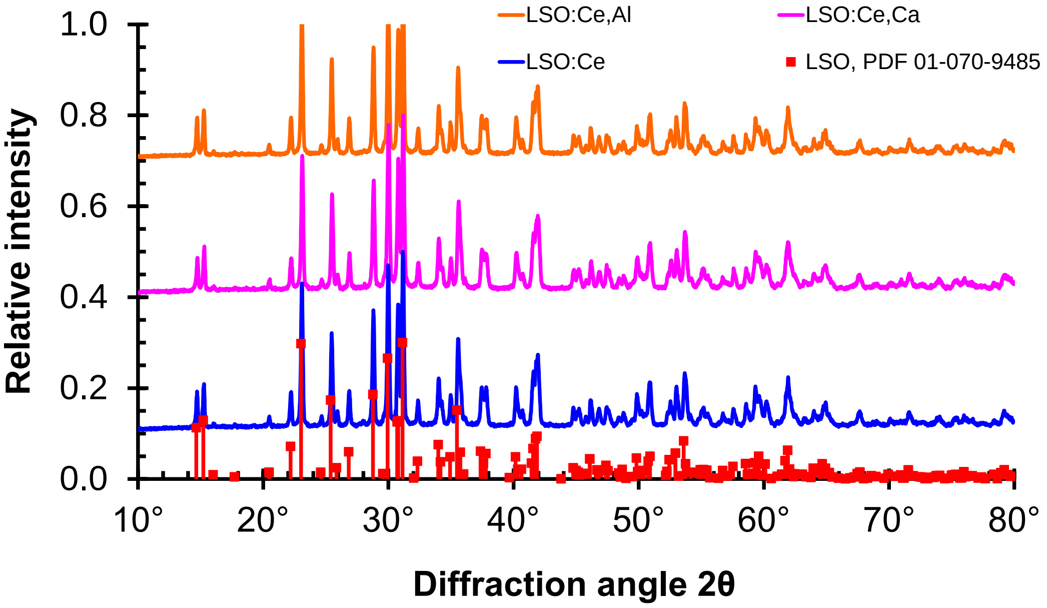

Fig. SI1 – Diffraction patterns of LSO:Ce and co-doped samples

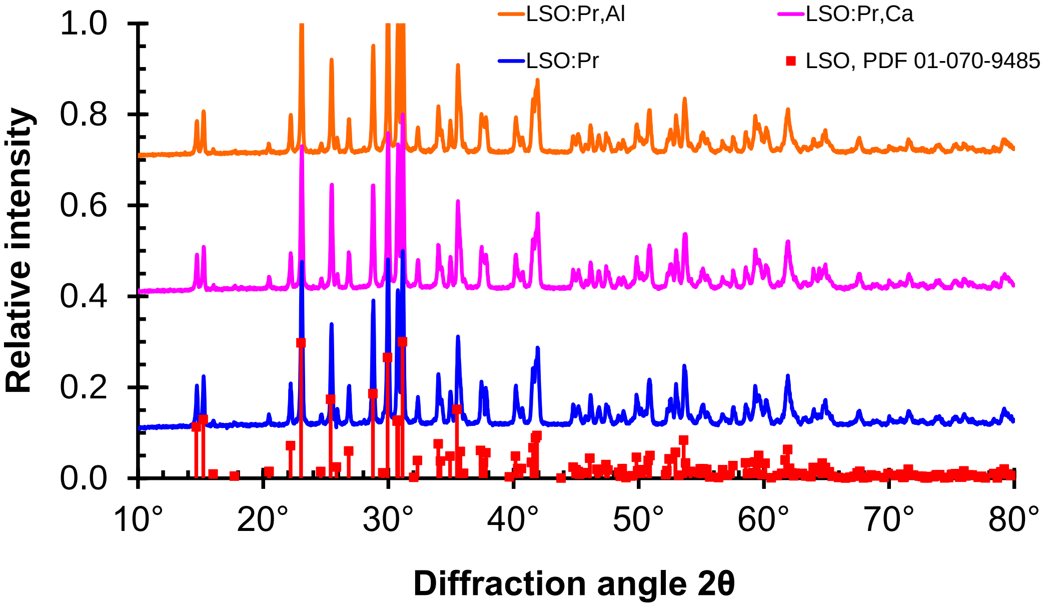

Fig. SI2 – Diffraction patterns of LSO:Pr and co-doped samples

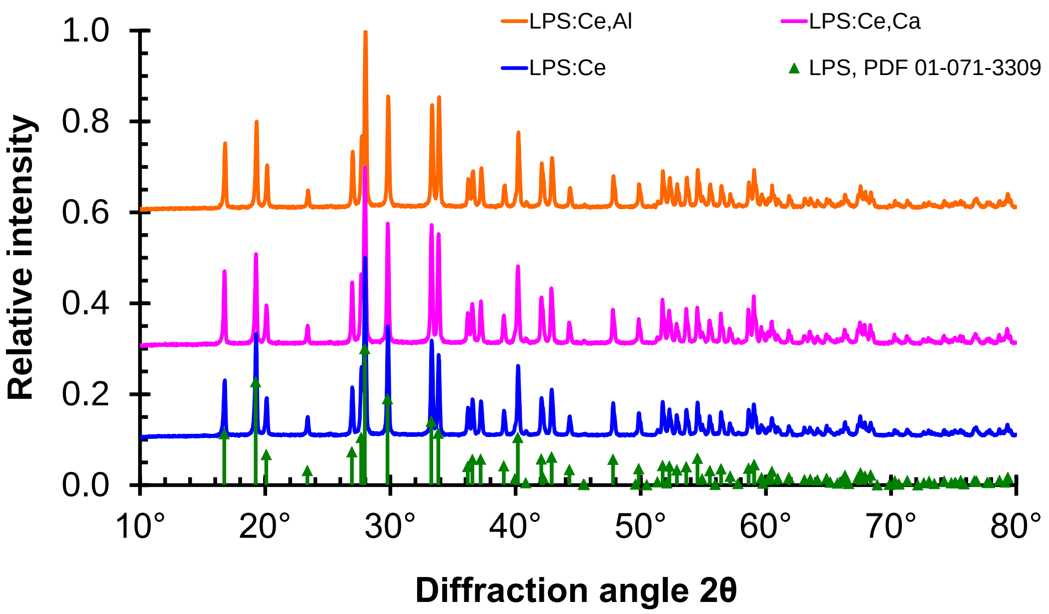

Fig. SI3 – Diffraction patterns of LPS:Ce and co-doped samples

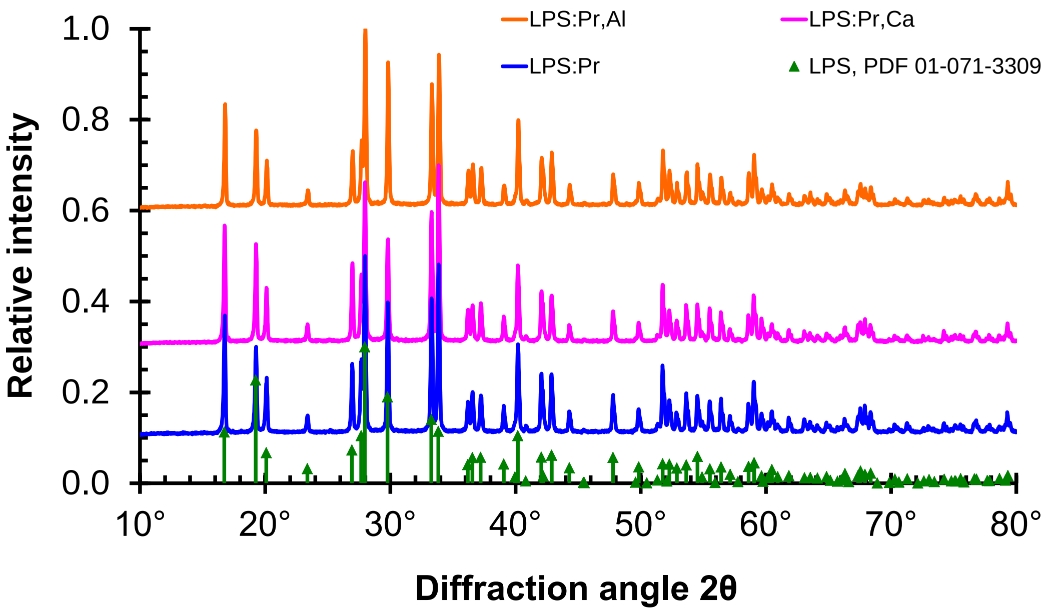

Fig. SI4 – Diffraction patterns of LPS:Pr and co-doped samples
